# Development and validation of a bedside risk score for MRSA among patients hospitalized with complicated skin and skin structure infections

**DOI:** 10.1186/1471-2334-12-154

**Published:** 2012-07-11

**Authors:** Marya D Zilberberg, Paresh Chaudhari, Brian H Nathanson, Rebecca S Campbell, Matthew F Emons, Suzanne Fiske, Harlen D Hays, Andrew F Shorr

**Affiliations:** 1 EviMed Research Group, LLC, Goshen, MA, USA; 2 School of Public Health and Health Sciences, University of Massachusetts, Amherst, MA, USA; 3 Astellas Pharma US, Inc, Deerfield, IL, USA; 4 OptiStatim LLC, Longmeadow, MA, USA; 5 Cerner Corporation, Beverly Hills, CA, USA; 6 Washington Hospital Center, Washington, DC, USA

**Keywords:** Skin infection, Prediction rule, Clinical decision, MRSA, Hospitalization

## Abstract

**Background:**

Methicillin-resistant *Staphylococcus aureus* (MRSA) is a frequent cause of complicated skin and skin structure infections (cSSSI). Patients with MRSA require different empiric treatment than those with non-MRSA infections, yet no accurate tools exist to aid in stratifying the risk for a MRSA cSSSI. We sought to develop a simple bedside decision rule to tailor empiric coverage more accurately.

**Methods:**

We conducted a large multicenter (N=62 hospitals) retrospective cohort study in a US-based database between April 2005 and March 2009. All adult initial admissions with ICD-9-CM codes specific to cSSSI were included. Patients admitted with MRSA vs. non-MRSA were compared with regard to baseline demographic, clinical and hospital characteristics. We developed and validated a model to predict the risk of MRSA, and compared its performance via sensitivity, specificity and other classification statistics to the healthcare-associated (HCA) infection risk factors.

**Results:**

Of the 7,183 patients with cSSSI, 2,387 (33.2%) had MRSA. Factors discriminating MRSA from non-MRSA were age, African-American race, no evidence of diabetes mellitus, cancer or renal dysfunction, and prior history of cardiac dysrhythmia. The score ranging from 0 to 8 points exhibited a consistent dose–response relationship. A MRSA score of 5 or higher was superior to the HCA classification in all characteristics, while that of 4 or higher was superior on all metrics except specificity.

**Conclusions:**

MRSA is present in 1/3 of all hospitalized cSSSI. A simple bedside risk score can help discriminate the risk for MRSA vs. other pathogens with improved accuracy compared to the HCA definition.

## Background

Although rates of serious infections with pathogens such as methicillin-resistant *Staphylococcus aureus* (MRSA) have been rising over recent years [1], there is encouraging news from the Centers for Disease Control and Prevention (CDC) indicating that this rise may be abating for healthcare-associated pathogens, though not for community-acquired ones [2]. Much of the emergent resistance can be attributed to selection pressures created by overuse of broad-spectrum antibiotics [3,4]. Empiric treatment of an infection becomes a balancing act for physicians concerned with containing the spread of resistance vs. not covering broadly enough, impacting clinical response and potentially outcomes [5-11].

Complicated skin and skin structure infections (cSSSI) are a common reason for hospitalization. *S. aureus*-related cellulitis hospitalizations, for example, have risen 4-fold in a recent 6-year period to over 90,000 discharges [1], and MRSA is the cause of cellulitis in 15% of all cSSSI cases [12]. While there is indirect evidence that inappropriate choice of empiric therapy may impact clinical outcomes among patients hospitalized with cSSSIs [12], this has not been confirmed broadly [11]. However, what does appear to be affected by the initial antibiotic choice is the duration of hospitalization [11], an outcome of utmost importance given the fiscal constraints in the healthcare system. For this reason, an early risk stratification tool for the presence of MRSA infection would be useful because a) prompt targeted treatment would balance appropriate spectrum coverage with concerns for resistance emergence and b) conventional laboratory testing takes 48–72 h to yield culture results.

We set out to compare current epidemiology and outcomes of patients hospitalized with MRSA cSSSI to those hospitalized with non-MRSA. Based on these differences, we developed and validated a clinical decision tool to help guide appropriate antimicrobial treatment at presentation.

## Methods

### Data source

This retrospective cohort study used data collected from 62 hospitals in the *Health Facts* electronic medical record database (Cerner Corporation, Kansas City, MO). *Health Facts* is a database built from hospitals’ comprehensive clinical records including pharmacy, laboratory, admission, and billing information from all affiliated patient care locations. All admissions, medication orders, and laboratory orders and collections are labeled with date and time. Hospital billing/registration and encounter data are integrated with clinical information relating to the drugs dispensed and the results of diagnostic laboratory testing. Cerner Corporation has established Health Insurance Portability and Accountability Act (HIPAA)–compliant operating policies and procedures using statistical methods to establish de-identification.

### Cohort selection

Included patients were at least 18 years of age, hospitalized between April 2005 and March 2009, and met our diagnostic criteria in one of two ways: 1) Admission had a primary diagnosis from Additional file 1: Appendix A **OR** 2) Admission had a secondary diagnosis from Additional file 1: Appendix A, a primary diagnosis of sepsis (International Classification of Diseases, Clinical Modification [ICD-9-CM] codes 020.2, 038.xx, 790.7, 995.91, or 995.92), and no other source of infection documented by ICD-9-CM code. Patients were also required to have both 1) collection of a blood or skin culture that was positive for bacteria and 2) treatment with any intravenous (IV) antibiotic within 48 h of presentation. The positive index culture had to contain at least one organism that was not considered to be a skin contaminant. Cultures of organisms considered to be skin contaminants (*Corynebacterium/diphtheroids, Staphylococcus epidermidis* or other coagulase-negative *Staphylococcus* sp., *Propionibacterium, Streptococcus viridians, Aerococcus* spp*, Bacillis* spp [except *B. anthracis*]) were excluded from the cohort. Other exclusions were pregnancy or complications of childbirth, major trauma, and inpatient admission within 30 days with primary or secondary diagnosis of cSSSI (Additional file 1: Appendix A).

### Measures

Microbiology results were prioritized to reduce the likelihood of colonizing microorganisms, though included patients could have polymicrobial infection. For patients with multiple culture results within the 48-hour timeframe, cultures from a skin source or venipuncture were prioritized over blood cultures drawn from a line, and certain sources of skin cultures with a high risk of contamination such as “swabs” were excluded. Lower extremity infections were identified using the 4th digit of the ICD-9-CM diagnosis code and/or the anatomic location of the skin culture, if present. Healthcare-associated (HCA) infection was defined based on the presence of at least one of the following factors: 1) admission from a chronic care facility, or 2) hospitalization within the previous 180 days or recent outpatient surgery, or 3) hemodialysis within 90 days or end-stage renal disease (ESRD) diagnosis code, or 4) chronic dependence on mechanical ventilation. Impaired immune function, based on specific ICD-9-CM codes and/or medication use, was examined separately. Chronic comorbidities present during admission or 12 months prior were identified by ICD-9-CM codes; baseline (within 48 hours of admission) laboratory variables were also recorded. Organ dysfunction measures at or before the time of the index culture were designed to be consistent with a Sepsis-related Organ Failure Assessment (SOFA) score equal to 2 or greater [13]. These included cardiovascular, respiratory, hepatic, hematologic, and renal dysfunction. Unadjusted hospital mortality served as the primary endpoint, while length of stay (LOS) among survivors was the secondary outcome.

Based on statistical and clinical considerations, we built and explored the usefulness of a simple summary score to identify infection with MRSA vs. a non-MRSA organism.

### Analyses

Our aim was to develop a simple summary score to be used at the bedside to serve as a clinical decision tool to distinguish patients with MRSA from those with an infection with another pathogen (non-MRSA). To accomplish this, we first compared patients admitted with MRSA to those with non-MRSA with regard to baseline demographic, clinical and hospital characteristics. To test for differences between groups, we used t-tests for continuous variables and the chi square test or Fisher’s exact test for categorical variables. When constructing the summary scores, potential covariates were included in the models if they reached significance at alpha=0.2 in the univariate analyses, or if *a priori* they were determined to have clinical relevance (Additional file 1: Appendix B) [14]. Variables were included only if they were present on admission or could be known within the first 24 hours of admission. The rationale for the 24-hour window was that inappropriately targeted empiric antimicrobial coverage within this time frame is associated with worsened outcomes [5-11]. In the selection of laboratory variables, we only considered tests for which at least 95% of values were obtained within 24 hours of admission.

A split sample approach was used, whereby 80% of the patients were randomly selected for model development and the remaining 20% were used for model validation. To identify the most predictive variables of MRSA vs. non-MRSA infection, we constructed a series of backward stepwise logistic regression models on bootstrapped with replacement (simulated) datasets from the development set and created a frequency table indicating the number of times the eligible predictors entered these stepwise regression models. The variables appearing most frequently were then considered for inclusion in the final summary score [15]. The score was designed *a priori* to rely on no more than 5 predictors for ease of use in clinical practice (which compelled us to exclude highly predictive but rare variables from the final model). The final model’s predictors were included in a logistic regression model and the coefficients were fully standardized to a unit variance. The predictors in the summary score were then assigned integer-valued weights proportional to these standardized coefficients.

We calculated the prevalence of MRSA infection at every score value in the development cohort and compared these results to the prevalence in the validation cohort to assess calibration. A simple, overall classification rate is often misleading when the overall prevalence of a condition is either quite high or low. To better assess the predictive ability of the models, we calculated specificities and sensitivities at various threshold levels of the score(s) to derive likelihood ratios. With these statistics, we constructed Fagan’s nomograms to show a patient’s posterior probability of having MRSA if the score was above or below a particular threshold (i.e., being above a threshold level was analogous to a positive “test”) [16].

To augment usefulness at the bedside and to benchmark the score against the HCA risk factors, an additional assessment of score characteristics was done calculating sensitivity, specificity, positive and negative predictive values for each threshold score along the scale. These characteristics were then compared to the performance of the HCA definition as a way to discriminate the risk for MRSA from that for non-MRSA infection.

## Results

### Patient characteristics

Among the 7,183 patients hospitalized with cSSSI, MRSA was cultured in 2,387 (33.2%), with the remainder (n=4,796) infected with non-MRSA (Table 1). Compared to patients with non-MRSA, those with MRSA were younger, less likely to be Caucasian, more likely to be on the medical service, and had lower comorbidity burden. Additionally, fewer of them had any risk factors for HCA infection (MRSA 32.6%, non-MRSA 36.8%, p<0.001) or a history of immune suppression (MRSA 22.9%, non-MRSA 28.9%, p<0.001).

**Table 1:** Patient demographics and hospital characteristics

	**Total sample (N= 7183)**	**MRSA (N = 2387)**	**Non- MRSA (N = 4796)**	**P-Value**
	**Frequency (%)**	**Frequency (%)**	**Frequency (%)**	
**Age in years, mean (SD)**	56.5 (19.4)	51.5 (19.6)	59.0 (18.8)	<0.001
18-44	2066 (28.8%)	956 (40.1%)	1110 (23.1%)	
45-54	1285 (17.9%)	463 (19.4%)	822 (17.1%)	
55-64	1275 (17.8%)	335 (14%)	940 (19.6%)	
65-74	971 (13.5%)	229 (9.6%)	742 (15.5%)	
75-84	947 (13.2%)	258 (10.8%)	689 (14.4%)	
>85	639 (8.9%)	146 (6.1%)	493 (10.3%)	
**Gender**				
Male	3966 (55.2%)	1364 (57.1%)	2602 (54.3%)	0.013
Female	3216 (44.8%)	1022 (42.8%)	2194 (45.7%)	
Unknown	1 (0%)	1 (0%)	0 (0%)	
**Race**				
Caucasian	5314 (74%)	1709 (71.6%)	3605 (75.2%)	<0.001
African American	1507 (21%)	572 (24%)	935 (19.5%)	
Other/Unknown	362 (5%)	106 (4.4%)	256 (5.3%)	
**Insurance type**				
Commercial	963 (13.4%)	331 (13.9%)	632 (13.2%)	<0.001
Medicare	1741 (24.2%)	444 (18.6%)	1297 (27.0%)	
Medicaid	456 (6.3%)	161 (6.7%)	295 (6.2%)	
Self-paid	203 (2.8%)	86 (3.6%)	117 (2.4%)	
Other/Unknown	3820 (53.2%)	1365 (57.2%)	2455 (51.2%)	
**Patient type**				
Surgical	1983 (27.6%)	613 (25.7%)	1370 (28.6%)	0.010
Medical	5200 (72.4%)	1774 (74.3%)	3426 (71.4%)	
**Year of discharge**				
2005	875 (12.2%)	265 (11.1%)	610 (12.7%)	0.380
2006	1648 (22.9%)	552 (23.1%)	1096 (22.9%)	
2007	1935 (26.9%)	660 (27.6%)	1275 (26.6%)	
2008	2250 (31.3%)	750 (31.4%)	1500 (31.3%)	
2009	475 (6.6%)	160 (6.7%)	315 (6.6%)	
**Hospital Characteristics**				
Census region				
Northeast	2887 (40.2%)	818 (34.3%)	2069 (43.1%)	<0.001
Midwest	1897 (26.4%)	573 (24.0%)	1324 (27.6%)	
South	2123 (29.6%)	923 (38.7%)	1200 (25%)	
West	276 (3.8%)	73 (3.1%)	203 (4.2%)	
Urban setting (vs. rural)	7171 (99.8%)	2385 (99.9%)	4786 (99.8%)	0.358
**Hospital bed size**				
6-99	371 (5.2%)	166 (7.0%)	205 (4.3%)	<0.001
100-199	980 (13.6%)	316 (13.2%)	664 (13.8%)	
200-299	1751 (24.4%)	557 (23.3%)	1194 (24.9%)	
300-499	2116 (29.5%)	663 (27.8%)	1302 (27.1%)	
≥500	1965 (27.4%)	685 (28.7%)	1431 (29.8%)	
Teaching (vs. non-teaching)	4412 (61.4%)	1406 (58.9%)	3006 (62.7%)	0.002
**Charlson comorbidity index, mean (SD)**	1.8 (2.1)	1.5 (2.0)	2.0 (2.1)	<0.001
**Risk factors for healthcare-associated infection (any)**	2543 (35.4%)	777 (32.6%)	1766 (36.8%)	<0.001
Recent hospital discharge (within 180 days) or recent outpatient surgery	2,255 (31.4%)	702 (29.4%)	1,553 (32.8%)	0.011
Outpatient hemodialysis within 90 days or ESRD patient	591 (8.2%)	176 (7.4%)	415 (8.7%)	0.063
Admission from a chronic care facility	369 (5.1%)	105 (4.4%)	264 (5.5%)	0.046
Mechanical ventilation/Dependence on respirator	48 (0.7%)	12 (0.5%)	36 (0.8%)	0.224
**Immunosuppression Factors (any below), N (%)**	1931 (26.9%)	546 (22.9%)	1385 (28.9%)	<0.001
ESRD (current or prior)	868 (12.1%)	247 (10.3%)	621 (12.9%)	0.001
Received systemic corticosteroid/immunosuppressive/chemotherapy	590 (8.2%)	177 (7.4%)	413 (8.6%)	0.082
Leukemia, lymphoma, metastasis	388 (5.4%)	80 (3.4%)	308 (6.4%)	<0.001
Autoimmune diseases*	266 (3.7%)	75 (3.1%)	191 (4%)	0.076
Aplastic anemia and pancytopenia	144 (2.0%)	22 (0.9%)	122 (2.5%)	<0.001
Organ transplantation	116 (1.6%)	32 (1.3%)	84 (1.8%)	0.193
HIV/AIDS	55 (0.8%)	31 (1.3%)	24 (0.5%)	<0.001
Neutropenia	46 (0.6%)	6 (0.3%)	40 (0.8%)	0.004
Other immunosuppression factors**	17 (0.2%)	4 (0.2%)	13 (0.3%)	0.395
**Comorbidity (current or 12 months prior to admission unless noted), N (%)**				
Hypertension	3585 (49.9%)	1071 (44.9%)	2514 (52.4%)	<0.001
Diabetes mellitus by diagnosis code or medications	3440 (47.9%)	936 (39.2%)	2504 (52.2%)	<0.001
Diabetic lower extremity infection (current encounter)	1746 (24.3%)	458 (19.2%)	1288 (26.9%)	<0.001
Renal dysfunction at baseline	1348 (18.8%)	332 (13.9%)	1016 (21.2%)	<0.001
Heart failure	1221 (17.0%)	330 (13.8%)	891 (18.6%)	<0.001
Coronary artery disease	1139 (15.9%)	328 (13.7%)	811 (16.9%)	0.001
Complication of electronic internal device (ICD-9 Code = 996.6, current encounter)	1087 (15.1%)	259 (10.9%)	828 (17.3%)	<0.001
Chronic obstructive pulmonary disease	815 (11.3%)	250 (10.5%)	565 (11.8%)	0.100
Cardiac dysrhythmias (prior to index encounter)	569 (7.9%)	166 (7%)	403 (8.4%)	0.032
Acute coronary syndrome/angina	534 (7.4%)	144 (6%)	390 (8.1%)	0.001
Cancer	521 (7.3)	110 (4.6%)	411 (8.6%)	<0.001
Cerebrovascular disease	283 (3.9%)	81 (3.4%)	202 (4.2%)	0.093
Hematologic dysfunction at baseline	265 (3.7%)	87 (3.6%)	215 (4.5%)	0.096
Cirrhosis/chronic liver disease	198 (2.8%)	45 (1.9%)	153 (3.2%)	0.001
**Laboratory values, mean (SD)**				
Hematocrit (%)	36.7 (6.0)	37.4 (5.7)	36.4 (6.1)	<0.001
White blood cell count (k/mm^3^)	12.8 (8.4)	12.5 (8.3)	12.9 (8.4)	0.048
Serum creatinine (mg/dL)	2.0 (2.3)	1.7 (2.1)	2.1 (2.4)	<0.001
Platelet count (k/mm^3^)	266.8 (119.2)	272.2 (109.3)	264.1 (123.7)	0.015

* Rheumatoid arthritis, multiple sclerosis, psoriasis, Crohn's disease.

** Includes cystic fibrosis, bone marrow transplant, pulmonary tuberculosis, abnormalities of spleen.

**Abbreviations:***ESRD*, end-stage renal disease; *HIV/AIDS*, human immunodeficiency virus/acquired immune deficiency syndrome; *ICD-9*, International Classification of Disease; *MRSA*, methicillin-resistant *Staphylococcus aureus*; *SD*, standard deviation.

### Outcomes

Unadjusted hospital mortality was lower in the MRSA (3.0%) than the non-MRSA (4.7%) group, p<0.001. Similarly, the median hospital LOS was shorter in the MRSA (4.2 days) than in the non-MRSA (5.7 days) group, p <0.001.

### MRSA prediction score

**Figure 1:**
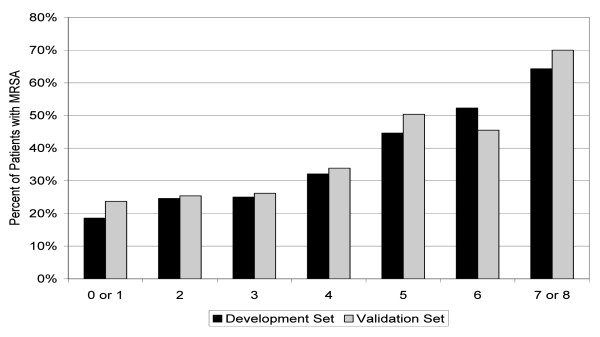
**Model calibration: proportion of patients with MRSA by the score value in development and validation cohorts.** MRSA=methicillin-resistant *S. aureus*.

The MRSA score was constructed on a random sample of 80% of the patients (development set, N=5,736) and validated on the remaining 20% (validation set, N=1,447). Factors differentiating MRSA infection from other organisms, based on the logistic regression, resulted in a score ranging from 0 to 8 points (Table 2). Those factors and their corresponding points were: age (range 0–3 points), African-American race (1 point), no evidence of diabetes mellitus (1 point), cancer (1 point) or renal dysfunction (1 point), and prior history of cardiac dysrhythmia (1 point). Increasing scores corresponded well to increased likelihood of MRSA infection in both development and validation cohorts (Figure 1).

**Table 2:** MRSA risk prediction score model

**Skin Infection, any site**						
Age	≤ 29	30–39	40–49	50–59	60+	Sub Score =
Points	2	3	2	1	0	
**Black Race**; If true add 1						
**No Evidence of Diabetes Mellitus** (DM) or Presence of Medication for the Treatment of DM; If true, add 1						
**No Evidence of Cancer** (eg, lymphoma, leukemia):						
If true, add 1						
**No Evidence of Renal Dysfunction**:						
No known outpatient Hemodialysis within 90 Days of Admission						
or						
known ESRD patient						
or						
current Serum Creatinine > 2 mg/dL; if true, add 1						
**Presence of Cardiac Dysrhythmias Prior to Index Encounter;** If present (if true); add 1						
						Total =

MRSA=methicillin-resistant *S. aureus*; ESRD=end-stage renal disease; WBC=white blood cells.

Table 3 presents the prevalence of actual MRSA isolates among patients with a particular score. While approximately 20% of all cases of MRSA had the score of 0–1, among those scoring 7–8 nearly 2/3 had the organism. Between these extremes, the score exhibited a consistent dose–response relationship.

**Table 3:** Prevalence of MRSA at different levels of the MRSA score in the dataset

**When the MRSA Score** **is →**	**0 or 1**	**2**	**3**	**4**	**5**	**6**	**7 or 8**	**Overall Rate**
Percent of Observed	19.9%	24.8%	25.3%	32.5%	45.8%	50.9%	65.4%	**33.2%**
Patients with MRSA								

MRSA=methicillin-resistant *S. aureus.*

We additionally examined the test characteristics of various score thresholds for detecting MRSA, and compared those to the HCA definition (Table 4). A MRSA score of 5 or higher turned out to be superior to the HCA classification in all characteristics, while that of 4 or higher was superior on all metrics except specificity. Using the threshold of 5 as an example for the score, if a clinician is most interested in excluding the probability of MRSA, the negative predictive value (the probability of scoring below 5 given that there was no MRSA present) was 72.3%. This represents a 12% relative improvement over the negative predictive value of the HCA definition (64.3%).

**Table 4:** Test characteristics for various thresholds of the MRSA score and the HCA risk factors on the validation set

**MRSA score (0 to 8)**	**Sensitivity**	**Specificity**	**Positive predictive value**	**Negative predictive value**	**Overall prevalence of patients with the score value or higher in the validation set**
2	95.2%	7.7%	35.3%	75.3%	93.3%
3	80.2%	30.9%	38.0%	74.7%	72.9%
4**	61.6%	58.6%	44.0%	74.3%	48.4%
5*	44.8%	75.9%	49.6%	72.3%	31.2%
6	17.0%	90.4%	48.3%	67.4%	12.2%
7 or 8	2.8%	99.4%	70.0%	65.9%	1.4%
HCA risk factors	32.0%	64.5%	32.3%	64.3%	34.3%

*MRSA score of **5** or higher is superior to HCA risk factors on all classification metrics.

**MRSA Score of **4** or higher is superior to HCA risk factors on all metrics but specificity.

MRSA=methicillin-resistant *S. aureus*; HCA=healthcare associated.

**Figure 2:**
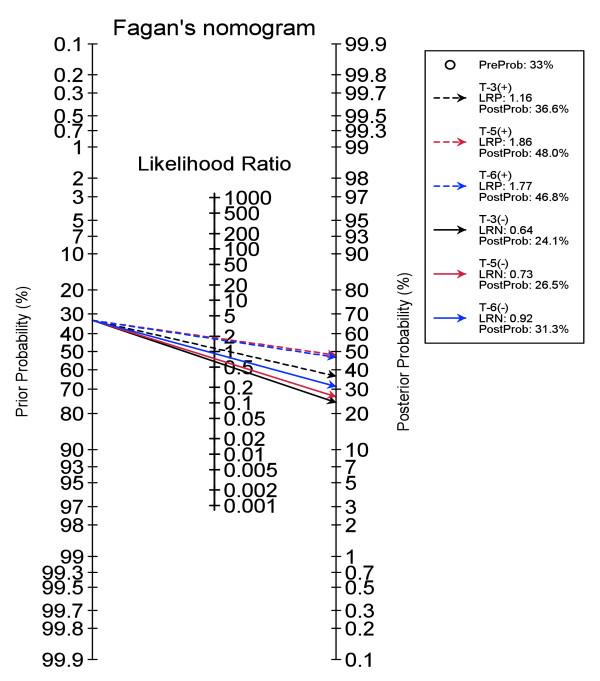
**Fagan’s Nomogram for the MRSA score.** MRSA=methicillin-resistant *S. aureus*; PreProb=pretest (prior) probability; PostProb=posterior probability; T=threshold score; LRP=likelihood ratio positive; LRN=likelihood ratio negative.

A Bayesian analysis was performed via Fagan’s nomogram on the validation set (Figure 2). The threshold classification results are based on the scores of 3 (T-3), 5 (T-5) and 7 (T-7). Using the pre-test probability of MRSA of 33% (the overall prevalence in our cohort), at a threshold score of 3 or higher the posterior probability of having MRSA is 34.5%. Similarly, for the score of 5 it becomes 43.0%, and for the score of 7 it is 48.2%. Alternatively, if the score is below 3, the posterior probability of having MRSA diminishes to less than 25%.

## Discussion

In this large multi-center cohort study, we have demonstrated that MRSA is present in one-third of all cSSSI patients hospitalized for their infection. There was a distinct age difference with regard to the risk for MRSA, wherein younger age groups were more likely to be infected with it relative to the older ones. Similarly, the burdens of comorbidities and risk factors for healthcare-associated infection were lower in the MRSA-infected group compared to those harboring non-MRSA. Commensurately, both hospital mortality and LOS among the MRSA-infected group were lower when compared to the non-MRSA group. Finally, we developed a bedside risk score to aid in the early identification of MRSA as a potential pathogen. Although improving somewhat the post-test probability of MRSA, the usefulness of the score may be limited by the high pathogen prevalence in the cohort.

Over the last decade, the increasing frequency of resistant organisms among persons presenting with infections from the community prompted a re-evaluation of the populations at risk for harboring such pathogens. Out of this effort, the HCA infection definition was born to signify a patient presenting from the community who is nevertheless put at risk for resistant pathogens by his/her ongoing contact with the healthcare system [15]. Some of the commonly acknowledged risk factors for HCA infection are recent hospitalization, immune suppression, need for hemodialysis and nursing home residence. Because the HCA risk factors were neither developed nor validated prospectively, several authors have expressed concern that such imprecise definition would lead to over-diagnosis of resistance, and thus overtreatment, with its sequelae of promoting further resistance development. For example, in a cohort of patients with HCA pneumonia (HCAP), Shorr et al. calculated the specificity of the HCAP risk factors for a resistant pathogen to be below 50% [17]. In an effort to sharpen accuracy, the investigators focused on identifying specific risk factors for resistant pathogens overall and for MRSA individually, with the view to developing a simple bedside risk score [17,18].

Our findings are important on several accounts. The risk factors we found that were strongly associated with the presence of a MRSA infection differed from the broadly used HCA definition. In fact, HCA was less likely to be present in the setting of MRSA than non-MRSA infection. Although this differs from other reports that found 2/3 of all MRSA cSSSIs to have underlying HCA risk factors, the discrepancy is likely due more to the lower prevalence of HCA overall in the current study rather than to fundamental microbiologic differences [19]. That is the prior study noted ~75% prevalence of HCA in the cohort of 717 patients admitted with a cSSSI, while in the current analysis it was only 35%. Given that the previous result came from a single-center analysis, the current data are more generalizable. Additionally, although the systematically derived score performed somewhat better than the consensus definition, it was far from perfect, thus shielding some, but not all, of those with susceptible pathogens from exposure to overly broad coverage. A similar finding was reported by Schreiber et al. in a group of patients with pneumonia and respiratory failure [20].

In the realm of cSSSI, the evidence-based guideline recommendations shed limited light on how to evaluate a patient’s risk for harboring MRSA [21]. Compounding this difficulty further is the emergence of distinct community-acquired genotypes: though they require expanded antimicrobial coverage, their risk factors diverge from the traditional HCA paradigm. Although we were unable to examine the genetic make-up of the organisms in our study, the finding that MRSA was more frequent in the younger age groups may be consistent with the high prevalence of CA-MRSA. This underscores the need for a better risk stratification tool in this area than the HCA definition alone can provide.

Our current effort extends the findings of previous investigators on the challenges of identifying resistant organisms upon presentation and prior to the availability of culture results. In our study, the HCA definition identified only 32.0% of patients with MRSA correctly, while mislabeling an additional 35.5% as having MRSA, thus potentially prompting a high volume of overuse of broad-spectrum coverage (Table 4). In contrast, different values for our MRSA score provide flexibility for balancing sensitivity with specificity, and, therefore the negative with the positive predictive values. Namely, if excluding the possibility of MRSA is of primary interest, then the MRSA score of 2 or higher provides a higher degree of confidence than the HCA definition, given its substantially higher negative predictive value. However, when accuracy in general is sought, it is clear that higher MRSA scores (e.g., 4 or more) possess better test characteristics than the HCA definition, thus leading to more targeted prescription and less potential misuse of antibiotics.

Our study has a number of strengths and limitations. As a large multicenter cohort representing over 60 US hospitals, it has a high degree of generalizability. At the same time, as a retrospective observational study it is subject to a number of threats to validity, including a selection bias. We mitigated the possibility of this by setting *a priori* cohort definitions and including all consecutive patients meeting our selection criteria. Given that we relied on administrative coding to define our cohort, the study is prone to some degree of misclassification. However, the same approach has been used widely by multiple investigators, and this type of misclassification is likely to be either minor or non-differential [11,12,22]. Finally, while we focused on predictors that had a strong association with MRSA and were common, it is possible that other variables not in our database would have yielded a more accurate model, though we note that the list of predictors examined was quite comprehensive.

## Conclusions

In summary, although our simple bedside score did improve on the specificity of the HCA definition, it left room for further improvement. Ultimately, what is needed is a bedside diagnostic tool that can identify MRSA and other resistant organisms rapidly and with a high degree of accuracy, so as to tailor treatment appropriately. Until such tools are available, and depending on the local patterns of resistance, empiric broad-spectrum coverage followed by prompt de-escalation in response to microbiology data may need to remain the standard of care.

## Competing interests

This study was funded by Astellas Pharma US (APUS), Inc, Deerfield, IL. Drs. Zilberberg and Shorr have received research and consulting funds from APUS. Dr. Chaudhari is an employee and a stockholder in APUS. Drs. Campbell and Emons, Ms. Fiske and Mr. Hays work for Cerner Corporation, which has received research funding from APUS. Dr. Nathanson works for OptiStatim, LLC, which has received funds from Cerner Corporation.

## Authors’ contributions

MDZ participated in the conception and design of the study, data interpretation and drafting and revising of the manuscript. PC participated in design of the study and revising of the manuscript. BHN participated in the design of the study, analyzed the data and revised the manuscript critically for intellectual content. RSC participated in the design of the study, study analyses and drafting of the manuscript. MFE participated in the conception and design of the study and revising of the manuscript. SF participated in data management and analysis. HDH participated in data management and analysis. AFS participated in the conception and design of the study, data interpretation and revising of the manuscript for important intellectual content. All authors read and approved the final manuscript.

## Disclosure

This study was funded by Astellas Pharma US (APUS), Inc, Deerfield, IL. Drs. Zilberberg and Shorr have received research and consulting funds from APUS. Dr. Chaudhari is an employee and a stock holder in APUS. Drs. Campbell and Emons, Ms. Fiske and Mr. Hays work for Cerner Corporation, which has received research funding from APUS. Dr. Nathanson works for OptiStatim, LLC, which has received funds from Cerner Corporation.

## Pre-publication history

The pre-publication history for this paper can be accessed here:

http://www.biomedcentral.com/1471-2334/12/154/prepub

## Supplementary Material

Additional file 1 **Appendix A.** Qualifying ICD-9-CM codes and Appendix B. List of variables tested in prediction models. Click here for file
