# Supplementary material for: Development and validation of a bedside risk score for MRSA among patients hospitalized with complicated skin and skin structure infections
Source: BMC Infect Dis. 2012 Jul 11;12:154. doi: 10.1186/1471-2334-12-154 (PMC3518172; doi:10.1186/1471-2334-12-154)
Supplement: Additional file 1 — Appendix A. Qualifying ICD-9-CM codes and Appendix B. List of variables tested in prediction models. [file 1471-2334-12-154-S1.docx]

**Appendix A. Qualifying ICD-9-CM codes**

| ICD-9 code | Description |
| --- | --- |
| 035 | Erysipelas |
| 680.x | Carbuncle and furuncle |
| 681.x | Cellulitis and abscess of finger and toe |
| 682.x | Other cellulitis and abscess |
| 683 | Acute lymphadenitis |
| 685 | Pilonidal cyst with abscess |
| 686.x, Except 686.1 | Other local infections of skin and subcutaneous tissues |
| 707.x | Chronic ulcer of skin |
| 910-917.1, 919.1 | Abrasion or friction burn with infection |
| 910-917.3, 919.3 | Blister, infected |
| 910-917.5, 919.5 | Insect bite, nonvenomous, infected |
| 910-917.7, 919.7 | Superficial foreign body, infected |
| 910-917.9, 919.9 | Other superficial injury of specified site, infected |
| 958.3 | Post-traumatic wound infection |
| 997.62 | Amputation stump infection (chronic) |
| 996.62 | Infection due to other internal vascular device, implant, and graft (excludes central lines) |

**Appendix B. List of variables tested in prediction models**

| **Demographics** |
| --- |
| Age |
| Gender |
| Race |
| Insurance type |
| **Patient Characteristics** **(Known within 24 hours of presentation)**  **All are current or prior to index encounter unless specified** |
| **Any immunocompromised condition** |
| Received systemic corticosteroid/immunosuppressive/chemotherapy |
| HIV/AIDS |
| Aplastic anemia and pancytopenia |
| End stage renal disease |
| Bone marrow transplant |
| Abnormalities of spleen |
| Organ transplantation |
| Pulmonary tuberculosis |
| Cystic fibrosis |
| Leukemia, lymphoma, metastasis |
| Neutropenia |
| Autoimmune diseases (RA, MS, Psoriasis, Crohn's disease) |
| **Any risk factor for nosocomial infection/HCFA infection** |
| Admission source=Transfer from SNF or hospital/other care facility |
| ER admission/discharge disposition of SNF/other care w/in 90 days |
| Recent hospital discharge (30 days) |
| Recent hospital discharge (90 days) |
| Recent hospital discharge (180 days) |
| Recent outpatient surgery |
| Dependence on respirator |
| Mechanical ventilation |
| Outpatient hemodialysis within 90 days or ESRD patient |
| **Comorbid conditions** |
| ACS/Angina |
| Coronary artery disease |
| Ischemic stroke/TIA (Prior to index encounter) |
| Other cerebrovascular disease |
| Heart failure |
| Cerebrovascular disease: Intracranial/intracerebral hemorrhage (Prior to index encounter) |
| Cardiac dysrhythmias - total (Prior to index encounter) |
| Lymphoma |
| Other cancer |
| Myeloma |
| Diabetes mellitus dx code |
| Diabetes mellitus dx code OR meds |
| Hypertension |
| COPD |
| Cirrhosis/chronic liver disease |
| Charlson comorbidity index |
| Hematologic dysfunction at baseline |
| Renal dysfunction at baseline |
| Complication Of electronic internal device (ICD-9 Code = 996.6) |
| Diabetic lower extremity infection (current encounter) |
| **Lab Values at Baseline** |
| Hematocrit |
| White blood cell count |
| Serum creatinine |
| Platelet count |
| **Hospital Characteristics** |
| Census region |
| Urban vs. Rural |
| Bed size |
| Teaching status |
